# BCL2-BH4 antagonist BDA-366 suppresses human myeloma growth

**DOI:** 10.18632/oncotarget.8513

**Published:** 2016-03-31

**Authors:** Jiusheng Deng, Dongkyoo Park, Mengchang Wang, Ajay Nooka, Qiaoya Deng, Shannon Matulis, Jonathan Kaufman, Sagar Lonial, Lawrence H. Boise, Jacques Galipeau, Xingming Deng

**Affiliations:** ^1^ Department of Hematology and Medical Oncology, Winship Cancer Institute, Emory University, Atlanta, USA; ^2^ Department of Radiation Oncology, Winship Cancer Institute, Emory University, Atlanta, USA; ^3^ The First Affiliated Hospital, Xi'An Jiaotong University, Xi'An, China

**Keywords:** small molecule BDA-366, BCL2, multiple myeloma, apoptosis

## Abstract

Multiple myeloma (MM) is a heterogeneous plasma cell malignancy and remains incurable. B-cell lymphoma-2 (BCL2) protein correlates with the survival and the drug resistance of myeloma cells. BH3 mimetics have been developed to disrupt the binding between BCL2 and its pro-apoptotic BCL2 family partners for the treatment of MM, but with limited therapeutic efficacy. We recently identified a small molecule BDA-366 as a BCL2 BH4 domain antagonist, converting it from an anti-apoptotic into a pro-apoptotic molecule. In this study, we demonstrated that BDA-366 induces robust apoptosis in MM cell lines and primary MM cells by inducing BCL2 conformational change. Delivery of BDA-366 substantially suppressed the growth of human MM xenografts in NOD-scid/IL2Rγ^null^ mice, without significant cytotoxic effects on normal hematopoietic cells or body weight. Thus, BDA-366 functions as a novel BH4-based BCL2 inhibitor and offers an entirely new tool for MM therapy.

## INTRODUCTION

Multiple myeloma (MM) is a common malignancy characterized by excessive proliferation of abnormal clonal plasma cells in the bone marrow (BM). In recent years, substantial progress has been made in the development of effective therapeutic approaches to treat MM, including autologous and allogeneic stem cell transplants, monoclonal antibodies, proteasome inhibitors, immunomodulatory drugs and recent chimeric antigen receptor-expressing T cells [1–7]. These approaches have significantly improved clinical outcomes and prolonged patient survival. However, due to the high phenotypic heterogeneity and plasticity of MM cells [8], none of these agents is curative, and the disease eventually relapses and becomes refractory to treatment [9]. Thus, the development of innovative therapeutics that can advance the field toward the cure of MM is very attractive.

B-cell lymphoma-2 (BCL2) protein is an anti-apoptotic molecule that is expressed at high levels in cancer cells including malignant plasma cell lines [10–12], and is related to cancer cell survival, early disease relapse and drug resistance [13–16]. As a survival factor, BCL2 protein contains four conserved domains: BCL2 homology 1 (BH1), BH2, BH3, and BH4, and a transmembrane domain that anchors the molecules in the mitochondria and endoplasmic reticulum (ER) membranes [17]. BH1, BH2 and BH3 domains together form a hydrophobic pocket with the BH3 domain buried inside (the BH3-binding pocket). This pocket facilitates protein-protein binding between BCL2 and the BH3 domain of pro-apoptotic BCL2 family members such as BIM (Bcl-2-like protein 11), BAX (BCL2-associated X protein) and BAK (BCL2 homologous antagonist killer), preventing BAX / BAK activation and consequent apoptosis through mitochondrial outer membrane permeabilization (MOMP) [18]. A panel of BH3 mimetics including ABT-737, ABT-263, ABT-199, AT-101, GX15-070 have been developed to disrupt the binding between BCL2 and pro-apoptotic BCL2 family members, and to block the anti-apoptosis function of the BCL2 molecule [19–22]. BH3-based BCL2 inhibitors are currently being evaluated in different phases of clinical trials [23, 24].

The N-terminal amphipathic helix BH4 domain (aa6-31) of BCL2 is known as a survival domain and is essential for the anti-apoptotic function of BCL2 protein [25, 26]. Removal of the BH4 domain by caspase-activated cleavage or genetic mutation converts BCL2 from an anti-apoptotic molecule into a death effector [27]. The BCL2 BH4 domain has been shown to directly bind and inhibit inositol 1,4, 5-trisphosphate receptors (IP3R) [28], which serve as the main intracellular Ca2^+^-release channel of the ER [29], a decoy peptide binding the BH4 domain by blocking the interaction between BCL2 and IP3R induces Ca^2+^-mediated apoptosis in MM cells [30]. We recently identified a small molecule, BDA-366, which is a member of the anthraquinone (1,4-diamino-9,10-anthraquinone) class, as a selective BCL2 BH4 antagonist. BDA-366 induces a conformational change in the BCL2 molecule that converts it to a death protein, and inhibits lung cancer growth *in vitro* and *in vivo* [31, 32]. In this study, we report that BDA-366 has potent anti-myeloma activity, inducing robust apoptotic death of MM cells *in vitro* and suppressing the growth of human MM cells *in vivo*.

## RESULTS

### BDA-366 suppresses human myeloma cell growth *in vitro*

**Figure 1:**
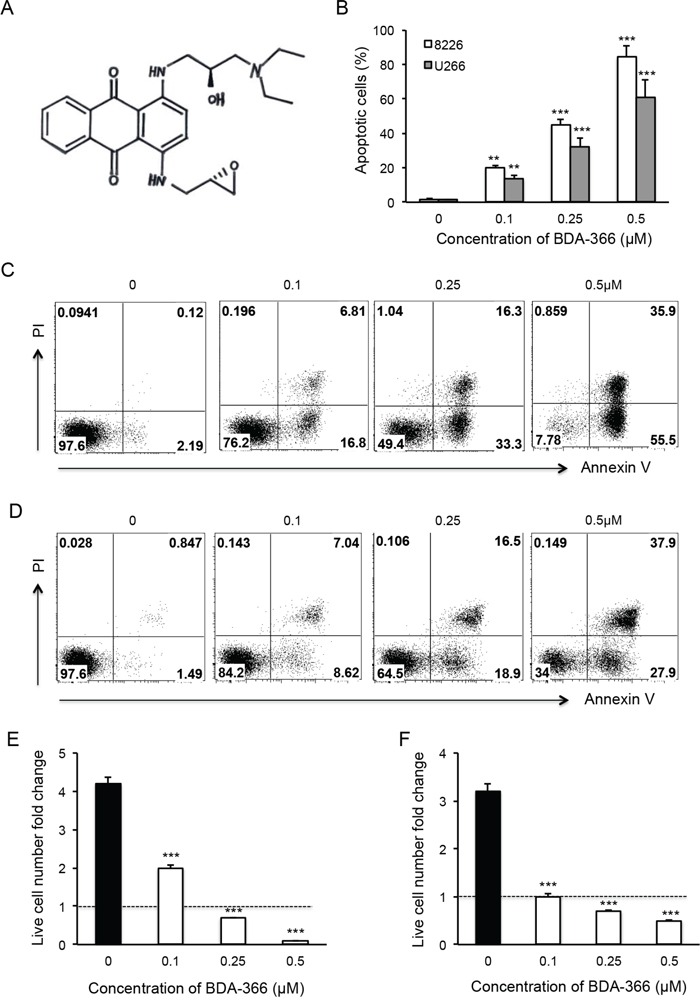
BDA-366 induces apoptosis in human myeloma cell lines Human MM cell lines RPMI8226 and U266 were treated with BDA366 in **A.** at increasing concentrations (0, 0.1, 0.25, 0.5μM) for 48hr in **B, C.** (RPMI8226) and **D.** (U266). Cells were harvested, stained with Annexin V and propidium iodide (PI), and subjected to FACS analysis. Apoptotic cells were gated on the Annexin V positive population. Annexin V^+^PI^−^ cells were early apoptotic cells, Annexin^+^PI^+^ cells were late apoptotic cells, and Annexin^−^PI^+^ cells were necrotic cells. (B) The percentages of apoptotic cells in the two treated cell lines are presented as mean± SEM. **E** and **F.** The absolute live cell number in each treatment was counted under a microscope with trypan blue staining. The fold change in RPMI8226 and U266 live cell number was calculated based on the initial cell number per well. Data shown represent three independent experiments.

BDA-366 is a small molecule antagonist that specifically binds the BCL2 BH4 domain, and has the ability to convert anti-apoptotic BCL2 into a pro-apoptotic death molecule [31]. To test the effect of the BH4 antagonist on myeloma cell growth, we treated human MM RPMI8226 and U266 cell lines with increasing concentrations of BDA-366 (Figure 1A) for 48 hours. FACS analyses of the treated MM cells showed that BDA-366 induced robust apoptosis in a dose-dependent manner in both RPMI8226 (Figure 1B and 1C) and U266 cells (Figure 1B and 1D). The percentages of apoptotic cells in RPMI8226 and U266 cell lines treated with 0.5μM of BDA-366 were 84.2% and 60.6% on average, respectively (Figure 1B), which were significantly higher than in cells treated with lower concentrations (i.e. 0.1μM or 0.25μM), or with DMSO control (Figure 1B). DMSO treatment had little apoptotic effect on the two cell lines (Figure 1B). Consequently, BDA-366 treatment markedly reduced the absolute live cell number in both RPMI8226 (Figure 1E) and U266 (Figure 1F) cell lines after 48-hour culture in comparison with DMSO control, with only 0.1 fold (RPMI8226, Figure 1E) and 0.5 fold (U266, Figure 1F) of the initial live cell numbers remaining in the groups treated with 0.5μM BDA-366. In contrast, DMSO treated groups showed an increase in absolute live cell number of more than 4-fold (RPMI8226, Figure 1E) and 3-fold (U266, Figure 1F) after 48-hour culture.

### BDA-366 induces conformational change and reduces the phosphorylation of BCL-2

**Figure 2:**
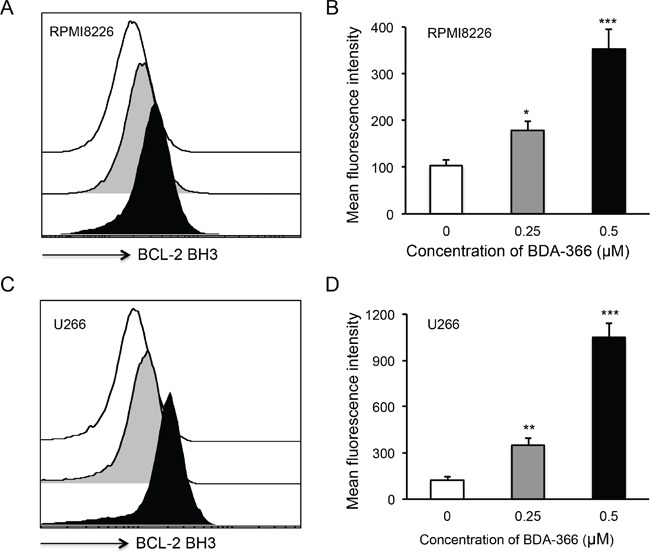
BDA-366 induces BCL2 conformational change in myeloma cell lines Human MM cell lines RPMI8226 in **A** and **B,** and U266 in **C** and **D.** were treated with BDA366 at concentrations of 0.25 and 0.5μM or with DMSO for 12hrs. Cells were harvested, intracellularly stained with anti-human BCL2 BH3- specific antibodies (1:100 dilution) before being subjected to FACS analysis. The histogram of BCL2 BH3 staining for RPMI8226 cells in (A) or U266 cells in (C) is a representative of three independent experiments, and the mean fluorescence intensity of BCL2 BH3 staining, as an indicator of BCL2 conformational change, in RPMI8226 cells in (B) or U266 in (D) cells was calculated from three independent experiments, and presented as mean± SEM.

**Figure 3:**
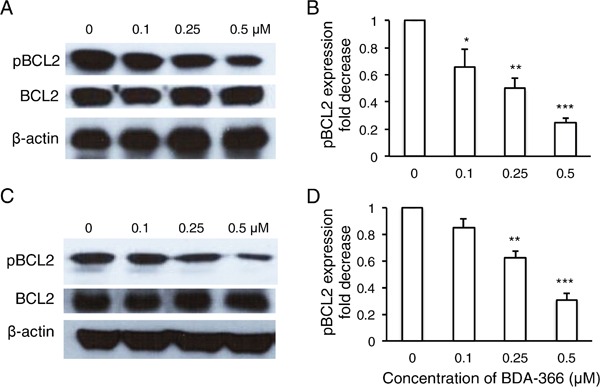
BDA-366 treatment suppresses BCL2 phosphorylation in myeloma cell lines RPMI8226 cells in **A** and **B.** and U266 cells in **C** and **D.** (10^6^/ml) were harvested after 8-hour BDA-366 treatment, and lysed with protein lysate buffer. Ten micrograms of protein in cell lysates from BDA-366 treated RPMI8226 cells in (A) or U266 in (C) was subjected to Western blot with anti-human pBCL2, BCL2, and anti-β-actin antibodies. Data shown in (A) and (C) are representative of three independent experiments. The fold change in pBCL2 expression induced by BDA-366 treatment in RPMI8226 cells in (B) or U266 cells in (D) was quantified based on BCL2 level and in comparison with untreated cells.

To examine whether BDA-366 induces BCL2 conformational change by exposure of the BCL2 BH3 domain in MM cells, we treated human RPMI8226 and U266 cells with BDA-366 at concentrations of 0.25 and 0.5μM, and stained the cells with anti-BCL2 BH3 domain specific antibody [31]. Flow cytometry assay demonstrated that BDA-366 treatment indeed induced the conformational change in BCL2 molecule that promotes the exposure of its BCL2 BH3 domain with significantly higher anti-BH3 fluorescence intensity in the treated RPMI8226 (Figure 2A–2B) and U266 (Figure 2C–2D) cells. It was previously demonstrated that phosphorylation of BCL2 at Ser70 is required to stabilize the anti-apoptotic activity of BCL2 and its function as a pro-survival molecule [33–35]. To examine whether BDA-366-induced BCL2 conformational change reduces BCL2 phosphorylation, we performed a Western blot on BDA-366-treated MM cells with an anti-pBCL2 (Ser70) specific antibody [31]. Western blotting showed that both RPMI8226 and U266 cells have a high baseline level of BCL2 phosphorylation (Figure 3). After BDA-366 treatments at concentration of 0.25 and 0.5μM, phosphorylation of BCL2 significantly decreased in both RPMI8226 (Figure 3A–3B) and U266 (Figure 3C–3D) cells, coincident with BDA-366-induced exposure of the BCL2 BH3 domain in the treated myeloma cells (Figure 2B and 2D).

### BDA-366 induces apoptosis in human primary myeloma cells

**Figure 4:**
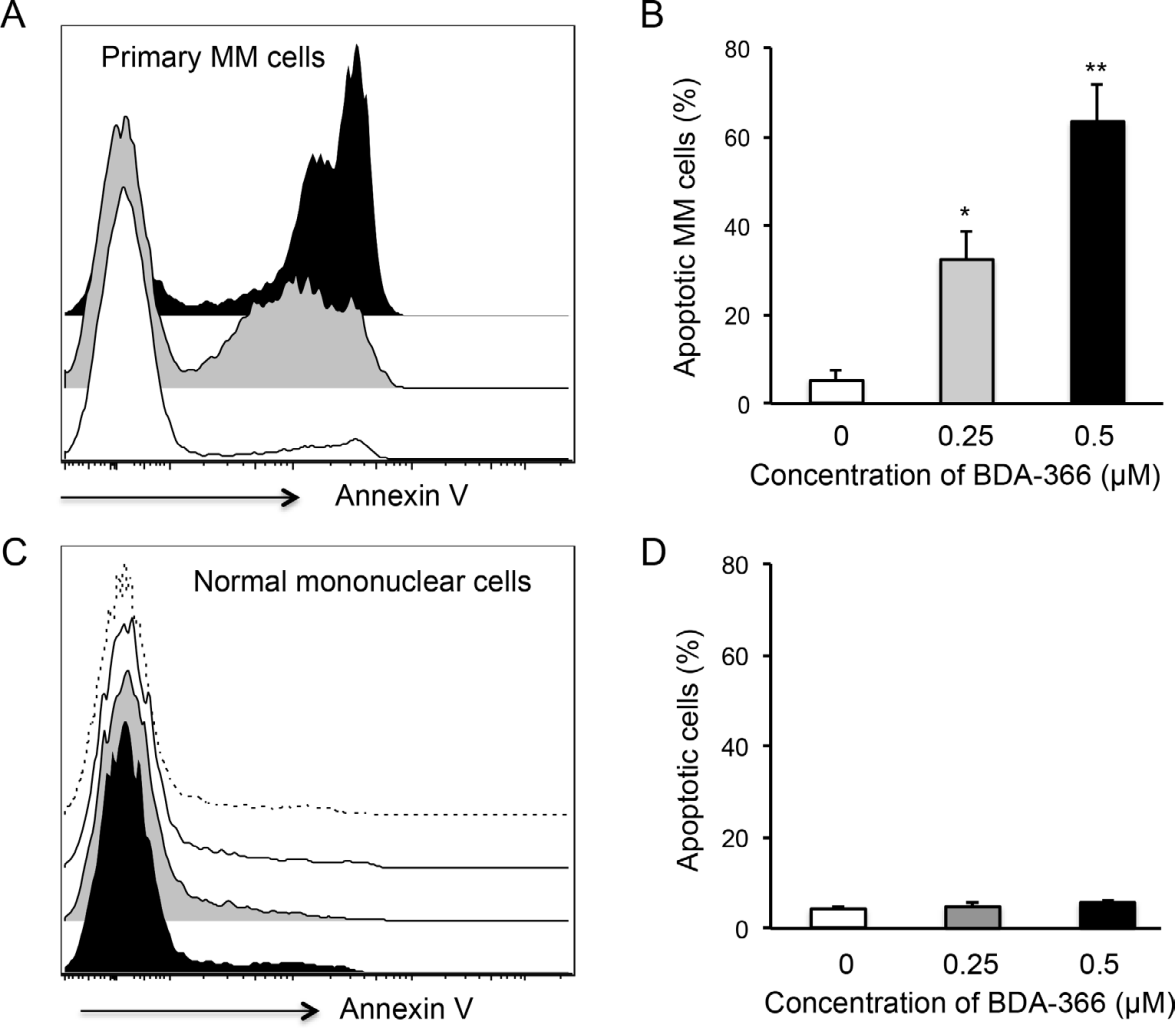
BDA-366 induces apoptosis in primary MM cells Mononuclear cells isolated from BM aspirates from MM patients (n=5) were treated with 0.25 (Gray) or 0.5μM (Black) BDA-366 or DMSO control (White) for 24hrs. After staining with anti-CD38, CD45 and CD138 antibodies, and Annexin V, the cells were subjected to FACS analysis. **A.** Apoptotic primary MM cells (CD45^−^CD38^+^CD138^+^) were gated on the Annexin^+^ population. **B.** The percentages of apoptotic MM cells from 5 patients are presented as mean± SEM. **C.** Apoptosis of bone marrow healthy mononuclear cells in response to BDA-366 treatment (0.25μM: Gray; 0.5 μM: Black) or DMSO (White); apoptotic cells were also gated on the Annexin-positive population. Cells cultured in medium only (dashed line) served as negative control. **D.** Percentages of apoptotic non-MM cells in the culture system were calculated and presented as mean± SEM.

**Figure 5:**
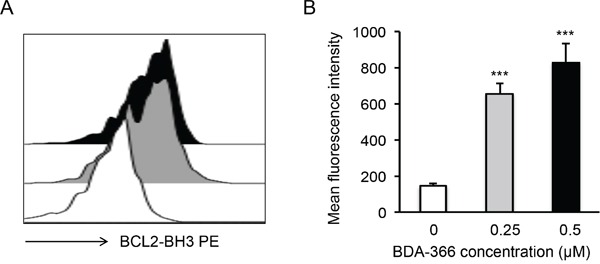
BDA-366 induces BCL2 conformational change in primary MM cells **A.** Primary MM cells (5×10^4^ cells/ml) from patients were treated with 0.25μM (Gray) or 0.5μM (Black) BDA-366, or with DMSO (White) for 12hrs. The cells were then stained with anti-BCL2 BH3 domain-specific antibodies and subjected to FACS analysis. Data shown are representative of five independent experiments (5 patients). **B.** The mean fluorescence intensity of BCL2 BH3 domain exposure in the treated primary MM cells from 5 patients was calculated and presented as mean± SEM.

To test the effect of the small molecule BDA-366 as a BCL2 BH4 antagonist on the apoptosis of primary myeloma cells, we treated the whole bone marrow (BM) cells harvested from myeloma patients with BDA-366. The patients were either untreated or had relapsed/refractory disease, with varying genetic backgrounds (Table 1). Primary myeloma cells were gated on the CD45^−^CD38^+^CD138^+^ population. FACS analyses of the treated BM cells demonstrated that BDA-366 treatment at concentrations of both 0.25 and 0.5 μM robustly induced the apoptosis of primary myeloma cells within a 24-hour culture period (Figure 4A), with 32.4% and 63.4% apoptotic cells on average respectively (Figure 4B). In control primary myeloma cells without BDA-366 treatment, there was a baseline level of 4.7% apoptotic cells. In non-MM cells, BDA-366 treatment did not cause significant apoptotic death in comparison with control treatment with DMSO (Figure 4C–4D). To examine whether the BH4 antagonist could also induce BCL2 conformational change by exposure of the BH3 domain in primary myeloma cells, we stained BDA-366-treated BM MM cells with anti-BCL2 BH3 domain-specific antibodies. FACS analysis of CD45^−^CD38^+^CD138^+^ cells showed that BDA-366 treatment at concentrations of 0.25 or 0.5μM indeed induced BCL2 conformational change in primary myeloma cells from patients, and caused the exposure of the BH3 domain (Figure 5A–5B).

**Table 1:** Patient characteristics

Sample	Sex	Age	Disease	ISS stage	CTG	FISH	Prior lines	LEN Ref	BTZ Ref	CFZ Ref	POM Ref
MM1374	M	71	Myeloma	1	46, XY	+IgH; +1q, monosomy 13	5	Yes	Yes	No	Yes
MM1377-2	F	63	Myeloma	3	46, XX	+ 1q, t(11;14), monosomy 13	6	Yes	Yes	Yes	Yes
MM1411	F	67	PPCL	1	39-44, XX	+IgH; +1q, monosomy 13, del 17p	0	No	No	No	No
MM1413	M	61	Myeloma	2	46, XY	+ IgH; +1q, monosomy 13, trisomy 3, 9, t(4;14)	0	No	No	No	No
MM1426	F	68	EMD	1	50, XX	+IgH; +1q, monosomy 13, trisomy 3, 9, t(4;14)	4	No	Yes	No	No

PPCL: primary plasma cell leukemia; EMD: extramedullary myeloma; M: Male; F: Female; ISS: International Staging System; CTG: cytogenetics; FISH: Flourescent in-situ hybridization; LEN: lenalidomide; BTZ: bortezomib; CFZ: carfilzomib; POM: pomalidomide; Ref: refractory.

### Administration of BDA-366 inhibited human myeloma cell growth in vivo

**Figure 6:**
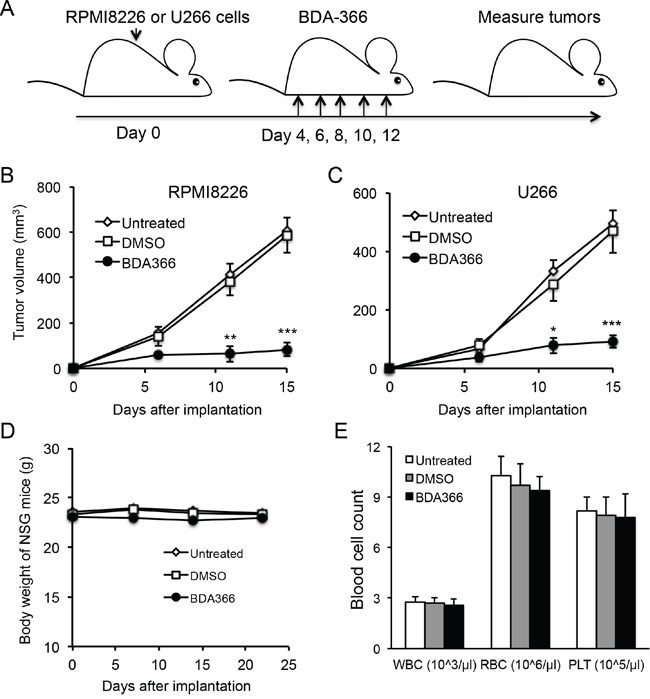
BDA-366 inhibits the growth of human MM cells in NSG mice **A.** Human myeloma cell lines RMPI8226 or U266 (5×10^6^ cells/mouse) were subcutaneously implanted into NSG immune deficient mice (n=5 per group). Each mouse received five doses of BDA-366 (10mg/Kg body weight), DMSO, or no treatment. **B** and **C,** After implantation, RPMI8226 in (B) or U266 in (C) myeloma tumors were measured, and tumor sizes in the three groups of mice were calculated and presented as mean± SEM. Data shown are from three independent experiments. **D** and **E,** To examine the cytotoxicity of the small molecule *in vivo*, naïve NSG mice were treated with five doses of BDA-366 or DMSO on day 0, 2, 4, 6 and 8, or received no treatment. The body weight in (D) was monitored with a digital scale. White blood cells (WBC), red blood cells (RBC) and platelets (PLT) in peripheral blood were collected from the NSG mice on day 23 before sacrifice, and profiled on an automatic blood cell counter. Data in (E) were from five mice per group, and presented as mean± SEM.

To test whether the efficacy of BDA-366 in the suppression of human MM cell growth *in vivo* reflects that seen *in vitro*, we implanted RPMI8226 or U266 myeloma cells into NSG mice, and treated the mice with BDA-366 at a dose of 10mg per kg mouse weight by intraperitoneal injection every 2 days from day 4-12 (Figure 6A). Measurement of myeloma tumors showed that delivery of BDA-366 into the mice significantly inhibited both RPMI8226 (Figure 6B) and U266 (Figure 6C) myeloma cell growth *in vivo*. Control DMSO treatment had no effect on the tumor growth (Figure 6B–6C). In parallel, we also assessed the cytotoxicity of BDA-366 in naive NSG mice following 5 treatment doses. Monitoring mouse body weight showed that BDA-366 treatment had no significant toxic side effects in the mice; the three groups of mice treated with the antagonist, DMSO, or no treatment had similar weights on day 23 (Figure 6D). Peripheral blood cell counts demonstrated that there were similar levels of white blood cells, red blood cells and platelets in the blood collected from the three groups of mice (Figure 6E), confirming that BDA-366 treatment had no significant toxic side effects on the murine hematopoietic system at a therapeutic dose.

## DISCUSSION

In this study, we demonstrate that the non-peptidic small molecule BDA-366 functions as a novel BCL2 inhibitor and effectively induces robust apoptotic death of human MM cells. To our knowledge, this is the first report that BH4 antagonist-induced BCL2 conformational change leads to the suppression of MM growth.

The mechanistic action of current BH3 mimetics for cancer therapy is designed to block the binding between BCL2 and BIM, BAX and BAK. Utilizing BDA-366 as a BH4-based BCL2 inhibitor to treat MM is distinct from this BH3 mimetics strategy, since BDA-366 not only blocks the activities of the BH4 domain, but also changes the conformation of BCL2 in the hydrophobic groove. During BCL2 conformational change, the hydrophobic pocket undergoes a large-scale realignment, causing the exposure of the BH3 domain [36], which can be detected by a BCL2 BH3-specific antibody [31]. Indeed, using anti-BCL2 BH3-specific antibodies, we were able to show this conformational change in BCL2 in MM cells treated with BDA-366, confirming the induction of BCL2 conformational change by the BH4 antagonist.

BCL2 phosphorylation at Ser70 promotes tumor cell survival [33, 35]. In this study, we found that BDA-366 treatment significantly reduced BCL2 phosphorylation at Ser70, indicating an additional mechanistic effect of BDA-366 on BCL2. BDA-366 was previously shown to disrupt the noncanonical BCL2-BH4-BAX interaction, inhibit the binding of BCL2 with IP3R and enhance Ca^2+^ release and apoptosis in tumor cells [31]. BCL2 BH4 domain also binds to calcineurin, CED-4, HIF-1α, c-Myc, paxillin, Raf-1, Ras and VDAC, promoting tumorigenesis or tumor survival and contributing to both anti-apoptosis and pro-tumorgenesis activities of BCL2 [37]. BDA-366-induced suppression of MM growth may also result from the inhibition of those functions of the BH4 domain.

MM is considered a heterogeneous disease in terms of malignant plasma cell subclones and its sensitivity to inhibition by anti-apoptotic BCL2 family proteins [38]. Using a BH3-profiling method [39], RPMI8226 cells were shown to be sensitive to the BCL2 inhibitor ABT-737 (a BCL2 BH3 mimetic), but insensitive to ABT-199, while U266 cells were resistant to the BH3 mimetics, since ABT-737 treatment had no significant suppressive effect on U266 cells [38]. In this study, we observed that RPMI8226 cells were very sensitive to BDA-366 treatment in cell culture. However, we also found that BDA-366 treatment potently induced apoptosis in U266 cells, suggesting that the BH4 antagonist employs distinct actions in malignant plasma cells compared to the BH3-based mimetics such as ABT-737 and ABT-199. BDA-366-induced robust apoptosis in primary myeloma cells from untreated patients or patients with relapsed/refractory myeloma with varying genetic background further indicates that BDA-366 as a novel BCL2 BH4 antagonist possesses potent therapeutic effect for relapsed/refractory myeloma. We also evaluated the anti-MM effect of BDA-366 in NSG immune deficient mice xenografted with RPMI8226 or U266 cells. Consistent with our *in vitro* results, BDA-366 treatment for 8 days efficiently suppressed human MM growth in the mice. Analyses of cytotoxicity of BDA-366 revealed that the BH4 antagonist had minimal side effects in terms of both body weight and the hematopoietic cellular system in NSG mice. Collectively, our data demonstrate that BDA-366, as a novel BH4-based BCL2 inhibitor and an inducer of BCL2 conformational change could offer an entirely new tool for MM therapy.

## MATERIALS AND METHODS

### BDA-366

Small molecule BDA-366 (NSC639366) [31] was obtained from the Drug Synthesis and Chemistry Branch, Developmental Therapeutic Program, Division of Cancer Treatment and Diagnosis, National Cancer Institute (Bethesda, MD), dissolved in DMSO as stock and diluted with PBS for *in vitro* and *in vivo* experimental use.

### Cell culture

Human myeloma cell lines RPMI8226 and U266 (obtained from the American Type Culture Collection) were cultured and maintained in RPMI-1640 medium (Hyclone, USA) supplemented with 10% heat-inactivated fetal bovine serum (FBS) (Gibco, USA) and penicillin / streptomycin antibiotics at 37°C in a 5% CO_2_ incubator. For cell apoptosis assay, U266 or 8226 cells (10,000 cells/well) were cultured in 96-well plates with 200ul/well of RPMI-1640 medium in the presence of different concentrations of BDA-366 (0, 0.1, 0.25, 0.5μM) for 48 hours.

### Patient sample processing

BM aspirates from patients with MM (n=5) (Table 1) were diluted to 20ml with 1× PBS, and processed as previously described [38]. White blood cells in the marrow were isolated with lymphocyte separation medium (Mediatech Inc., Manassas, VA), and cultured (10^6^ cells/ml) in complete RPMI medium supplemented with BDA-366 (0, 0.25, 0.5μM) for 24 hours. Informed consent was obtained from all human subjects. The use of human samples was approved by the Institutional Review Board of Emory University.

### Western blot

RPMI8226 or U266 cells (10^6^/ml) were harvested 8 hours after BDA-366 treatment (0, 0.1, 0.25, 0.5μM), and washed with cold PBS. Total proteins were extracted from the cells lysed with protein lysate buffer supplemented with protease and phosphatase inhibitors as previously described [40]. The proteins were separated on 10% SDS polyacrylamide gels and electrophoretically transferred to polyvinylidene difluoride membrane (Millipore, USA). The membranes were treated with rabbit anti-pBCL2 (Ser70), BCL2 (1:100), and anti-β-actin antibodies (1:1000 dilution) (Cell Signaling Technology, USA); then incubated with HRP-conjugated secondary antibodies before being subjected to enhanced chemiluminescent (ECL) detection on an ECL machine (Pierce, USA). The blots were scanned and the band density was measured using the Quantity One imaging software.

### Flow cytometry

BDA-366 treated RPMI8226 or U266 cells (10,000 cells/well) were harvested after 48-hour culture. Apoptotic cell fractions were assessed with Annexin-V FITC and propidium iodide staining kit following the manufacturer's instruction (Invitrogen, USA). Alternatively, BDA-366 treated BM cells from MM patients were stained with anti-human CD38 (PE), CD45 (APC-Cy7), and CD138 (APC) antibodies (1:100 dilution) (BD Bioscience, San Jose, CA), and Annexin V (FITC). Primary myeloma cells were gated on CD45^−^CD38^+^CD138^+^ cells; and apoptotic cells were gated on Annexin V as previously described [41]. In addition, RPMI8226, U266 or primary MM cells treated with BDA-366 for 12 hours were harvested and intracellularly stained with anti- BCL2 BH3 domain-specific antibody (Abgent, San Diego, CA) [31]. All stained cells were subjected to fluorescence-activated cell sorting (FACS) analyses on a BD Canto Flow Cytometer. All FACS data were analyzed with FlowJo 9.1 software.

### Blood counting

Peripheral blood was collected from immune deficient NOD-*scid* IL2Rgamma^null^ (NSG) mice directly to Microvette tubes containing EDTA-tripotassium salt (Sarstedt AG & Co, Nümbrecht, Germany), and subjected to blood cell counting on a Vet-ABC Animal Blood Counter (Scil, Gurnee, IL, USA) using mouse-specific software provided by the company.

### Myeloma xenografts in NSG mice

Human RPMI8226 or U266 myeloma cells (5×10^6^ cells/mouse) in 100μl of MatriGel solution (StemCell Technologies, Inc, Bedford, MA) were subcutaneously implanted in the right rear flanks of NSG immune deficient mice [42]. The mice were then treated with BDA-366 (10mg/Kg/day) on days 4, 6, 8, 10 and 12 after implantation. On day 15, myeloma tumors in the three groups of mice were measured with a digital caliper, and tumor size was calculated based on the length and width (micrometer) of the tumor: volume=length×(width)^2^/2. The mice used were all female (6-8 weeks old) purchased from Jackson Laboratory (Bar Harbor, ME), and maintained in compliance with an IACUC protocol approved by Emory University (Atlanta, USA).

### Statistical analysis

Data are shown as mean ± SEM. *P* values were calculated using the one-way analysis of variance test. *P* value of less than 0.05 was considered significant (*: *P*<0.05; **: *P*<0.01; *** *P*<0.001).

## References

[1] Matulis SM, Gupta VA, Nooka AK, Von Hollen H, Kaufman JL, Lonial S, Boise LH (2015). Dexamethasone treatment promotes Bcl-2-dependence in multiple myeloma resulting in sensitivity to Venetoclax. Leukemia.

[2] Laubach JP, Mitsiades CS, Mahindra A, Luskin MR, Rosenblatt J, Ghobrial IM, Schlossman RL, Avigan D, Raje N, Munshi NC, Anderson KC, Richardson PG (2011). Management of relapsed and relapsed/refractory multiple myeloma. J Natl Compr Canc Netw.

[3] Palumbo A, Anderson K (2011). Multiple myeloma. N Engl J Med.

[4] Nooka AK, Kastritis E, Dimopoulos MA, Lonial S (2015). Treatment options for relapsed and refractory multiple myeloma. Blood.

[5] Martino M, Recchia A, Fedele R, Neri S, Vincelli I, Moscato T, Gentile M, Morabito F (2016). The role of tandem stem cell transplantation for multiple myeloma patients. Expert Opin Biol Ther.

[6] Tai YT, Anderson KC (2015). Targeting B-cell maturation antigen in multiple myeloma. Immunotherapy.

[7] Maus MV, June CH (2014). CARTs on the road for myeloma. Clin Cancer Res.

[8] Kumar S, Kimlinger T, Morice W (2010). Immunophenotyping in multiple myeloma and related plasma cell disorders. Best Pract Res Clin Haematol.

[9] Pantani L, Brioli A, Tacchetti P, Zannetti BA, Mancuso K, Rocchi S, Martello M, Rizzello I, Terragna C, Zamagni E, Cavo M (2016). Current and emerging triplet combination therapies for relapsed and refractory multiple myeloma. Expert Rev Hematol.

[10] Hu W, Gazitt Y (1996). Bcl-2 plays a major role in resistance to dexamethasone induced apoptosis in multiple myeloma cell lines. Int J Oncol.

[11] Gazitt Y, Rothenberg ML, Hilsenbeck SG, Fey V, Thomas C, Montegomrey W (1998). Bcl-2 overexpression is associated with resistance to paclitaxel, but not gemcitabine, in multiple myeloma cells. Int J Oncol.

[12] Ong F, van Nieuwkoop JA, de Groot-Swings GM, Hermans J, Harvey MS, Kluin PM, Kluin-Nelemans JC (1995). Bcl-2 protein expression is not related to short survival in multiple myeloma. Leukemia.

[13] Boise LH, Gottschalk AR, Quintans J, Thompson CB (1995). Bcl-2 and Bcl-2-related proteins in apoptosis regulation. Curr Top Microbiol Immunol.

[14] Adams JM, Cory S (2007). The Bcl-2 apoptotic switch in cancer development and therapy. Oncogene.

[15] Puthier D, Pellat-Deceunynck C, Barille S, Robillard N, Rapp MJ, Juge-Morineau N, Harousseau JL, Bataille R, Amiot M (1999). Differential expression of Bcl-2 in human plasma cell disorders according to proliferation status and malignancy. Leukemia.

[16] Ailawadhi S, Miecznikowski J, Gaile DP, Wang D, Sher T, Mulligan G, Bryant B, Wilding GE, Mashtare T, Stein L, Masood A, Neuwirth R, Lee KP, Chanan-Khan A (2012). Bortezomib mitigates adverse prognosis conferred by Bcl-2 overexpression in patients with relapsed/refractory multiple myeloma. Leuk Lymphoma.

[17] Kelekar A, Thompson CB (1998). Bcl-2-family proteins: the role of the BH3 domain in apoptosis. Trends Cell Biol.

[18] Skommer J, Wlodkowic D, Deptala A (2007). Larger than life: Mitochondria and the Bcl-2 family. Leuk Res.

[19] Konopleva M, Contractor R, Tsao T, Samudio I, Ruvolo PP, Kitada S, Deng X, Zhai D, Shi YX, Sneed T, Verhaegen M, Soengas M, Ruvolo VR (2006). Mechanisms of apoptosis sensitivity and resistance to the BH3 mimetic ABT-737 in acute myeloid leukemia. Cancer Cell.

[20] Tse C, Shoemaker AR, Adickes J, Anderson MG, Chen J, Jin S, Johnson EF, Marsh KC, Mitten MJ, Nimmer P, Roberts L, Tahir SK, Xiao Y (2008). ABT-263: a potent and orally bioavailable Bcl-2 family inhibitor. Cancer Res.

[21] Kang MH, Reynolds CP (2009). Bcl-2 inhibitors: targeting mitochondrial apoptotic pathways in cancer therapy. Clin Cancer Res.

[22] Touzeau C, Dousset C, Le Gouill S, Sampath D, Leverson JD, Souers AJ, Maiga S, Bene MC, Moreau P, Pellat-Deceunynck C, Amiot M (2014). The Bcl-2 specific BH3 mimetic ABT-199: a promising targeted therapy for t(11;14) multiple myeloma. Leukemia.

[23] Kipps TJ, Eradat H, Grosicki S, Catalano J, Cosolo W, Dyagil IS, Yalamanchili S, Chai A, Sahasranaman S, Punnoose E, Hurst D, Pylypenko H (2015). A phase 2 study of the BH3 mimetic BCL2 inhibitor navitoclax (ABT-263) with or without rituximab, in previously untreated B-cell chronic lymphocytic leukemia. Leuk Lymphoma.

[24] Ng SY, Davids MS (2014). Selective Bcl-2 inhibition to treat chronic lymphocytic leukemia and non-Hodgkin lymphoma. Clin Adv Hematol Oncol.

[25] Huang DC, Adams JM, Cory S (1998). The conserved N-terminal BH4 domain of Bcl-2 homologues is essential for inhibition of apoptosis and interaction with CED-4. Embo J.

[26] de Moissac D, Zheng H, Kirshenbaum LA (1999). Linkage of the BH4 domain of Bcl-2 and the nuclear factor kappaB signaling pathway for suppression of apoptosis. J Biol Chem.

[27] Cheng EH, Kirsch DG, Clem RJ, Ravi R, Kastan MB, Bedi A, Ueno K, Hardwick JM (1997). Conversion of Bcl-2 to a Bax-like death effector by caspases. Science.

[28] Akl H, Vervloessem T, Kiviluoto S, Bittremieux M, Parys JB, De Smedt H, Bultynck G (2014). A dual role for the anti-apoptotic Bcl-2 protein in cancer: mitochondria versus endoplasmic reticulum. Biochim Biophys Acta.

[29] Rong YP, Bultynck G, Aromolaran AS, Zhong F, Parys JB, De Smedt H, Mignery GA, Roderick HL, Bootman MD, Distelhorst CW (2009). The BH4 domain of Bcl-2 inhibits ER calcium release and apoptosis by binding the regulatory and coupling domain of the IP3 receptor. Proc Natl Acad Sci U S A.

[30] Lavik AR, Zhong F, Chang MJ, Greenberg E, Choudhary Y, Smith MR, McColl KS, Pink J, Reu FJ, Matsuyama S, Distelhorst CW (2015). A synthetic peptide targeting the BH4 domain of Bcl-2 induces apoptosis in multiple myeloma and follicular lymphoma cells alone or in combination with agents targeting the BH3-binding pocket of Bcl-2. Oncotarget.

[31] Han B, Park D, Li R, Xie M, Owonikoko TK, Zhang G, Sica GL, Ding C, Zhou J, Magis AT, Chen ZG, Shin DM, Ramalingam SS (2015). Small-Molecule Bcl2 BH4 Antagonist for Lung Cancer Therapy. Cancer Cell.

[32] Chen G, Deng X (2015). Targeting Bcl2 in cancer. Oncoscience.

[33] Ruvolo PP, Deng X, May WS (2001). Phosphorylation of Bcl2 and regulation of apoptosis. Leukemia.

[34] Dai H, Ding H, Meng XW, Lee SH, Schneider PA, Kaufmann SH (2013). Contribution of Bcl-2 phosphorylation to Bak binding and drug resistance. Cancer Res.

[35] Low IC, Loh T, Huang Y, Virshup DM, Pervaiz S (2014). Ser70 phosphorylation of Bcl-2 by selective tyrosine nitration of PP2A-B56delta stabilizes its antiapoptotic activity. Blood.

[36] Petros AM, Medek A, Nettesheim DG, Kim DH, Yoon HS, Swift K, Matayoshi ED, Oltersdorf T, Fesik SW (2001). Solution structure of the antiapoptotic protein bcl-2. Proc Natl Acad Sci U S A.

[37] Liu Z, Wild C, Ding Y, Ye N, Chen H, Wold EA, Zhou J (2015). BH4 domain of Bcl-2 as a novel target for cancer therapy. Drug Discov Today.

[38] Morales AA, Kurtoglu M, Matulis SM, Liu J, Siefker D, Gutman DM, Kaufman JL, Lee KP, Lonial S, Boise LH (2011). Distribution of Bim determines Mcl-1 dependence or codependence with Bcl-xL/Bcl-2 in Mcl-1-expressing myeloma cells. Blood.

[39] Certo M, Del Gaizo Moore V, Nishino M, Wei G, Korsmeyer S, Armstrong SA, Letai A (2006). Mitochondria primed by death signals determine cellular addiction to antiapoptotic BCL-2 family members. Cancer Cell.

[40] Deng J, Yuan S, Pennati A, Murphy J, Wu JH, Lawson D, Galipeau J (2014). Engineered fusokine GIFT4 licenses the ability of B cells to trigger a tumoricidal T-cell response. Cancer Res.

[41] Rafei M, Deng J, Boivin MN, Williams P, Matulis SM, Yuan S, Birman E, Forner K, Yuan L, Castellino C, Boise LH, MacDonald TJ, Galipeau J (2011). A MCP1 fusokine with CCR2-specific tumoricidal activity. Mol Cancer.

[42] Chen S, Dai Y, Pei XY, Myers J, Wang L, Kramer LB, Garnett M, Schwartz DM, Su F, Simmons GL, Richey JD, Larsen DG, Dent P (2012). CDK inhibitors upregulate BH3-only proteins to sensitize human myeloma cells to BH3 mimetic therapies. Cancer Res.

